# Rosmarinic acid suppresses adipogenesis, lipolysis in 3T3-L1 adipocytes, lipopolysaccharide-stimulated tumor necrosis factor-α secretion in macrophages, and inflammatory mediators in 3T3-L1 adipocytes

**DOI:** 10.1080/16546628.2017.1330096

**Published:** 2017-06-07

**Authors:** Yehua Rui, Lingxia Tong, Jinbo Cheng, Guiping Wang, Liqiang Qin, Zhongxiao Wan

**Affiliations:** ^a^ Department of Nutrition and Food Hygiene, School of Public Health, Soochow University, Suzhou, PR China; ^b^ Department of Obstetrics and Gynecology, The Second Affiliated Hospital of Soochow University, Suzhou, PR China; ^c^ Laboratory Animal Center, Soochow University, Suzhou, PR China; ^d^ Jiangsu Key Laboratory of Preventive and Translational Medicine for Geriatric Disease, Soochow University, Suzhou, PR China

**Keywords:** Rosmarinic acid, lipolysis, adipogenesis, adipokines, adipocytes

## Abstract

**Background**: Rosmarinic acid (RA) is a natural phenol carboxylic acid with many promising biological effects. It may be a suitable candidate for improving obesity-related adipose tissue dysfunction.

**Objective**: We aimed to investigate the therapeutic use of RA as an anti-obesity agent by measuring its effects on adipogenesis, lipolysis, and messenger RNA (mRNA) expression of major adipokines in 3T3-L1 adipocytes; and its effects on lipopolysaccharide (LPS)-induced tumor necrosis factor-α (TNF-α) secretion in macrophages and inflammatory mediators in 3T3-L1 adipocytes incubated with macrophage-conditioned medium (MCM).

**Methods**: 3T3-L1 preadipocytes were used to explore how RA affects adipogenesis, as well as the involvement of phosphorylated extracellular signal-regulated kinase-1/2 (p-ERK1/2) and mothers against decapentaplegic homolog 3 (p-Smad3). 3T3-L1 preadipocytes were also differentiated into mature adipocytes to explore how RA affects basal and isoproterenol- and forskolin-stimulated lipolysis; and how RA affects key adipokines’ mRNA expression. RAW 264.7 macrophages were stimulated with LPS in the absence or presence of RA to explore RA’s effects on TNF-α secretion. MCM was collected and 3T3-L1 adipocytes were incubated with MCM to explore RA’s effects on interleukin-6 (IL-6), IL-1β, monocyte chemoattractant protein-1 (MCP-1), and RANTES mRNA expression.

**Results**: During the preadipocyte differentiation process, RA suppressed peroxisome proliferator-activated receptor-γ and CCAAT/enhancer binding protein-α, and activated p-ERK1/2 and p-Smad3; inhibition of adipogenesis by RA was partially restored following treatment with p-ERK1/2 and p-Smad3 inhibitors. In mature adipocytes, RA inhibited basal lipolysis; phosphodiesterase-3 inhibitor reversed this. RA also inhibited isoproterenol- and forskolin-stimulated glycerol and free fatty acid release, and the phosphorylation of hormone-sensitive lipase and perilipin. RA had no effects on leptin, adiponectin, resistin, or visfatin mRNA expression. RA suppressed TNF-α mRNA expression and secretion in LPS-stimulated RAW 264.7 macrophages; and reduced LPS-MCM-induced IL-6, IL-1β, MCP-1, and RANTES mRNA expression in 3T3-L1 adipocytes.

**Conclusions**: RA exerts inhibitory effects on adipogenesis, lipolysis, and inflammation. RA could be a promising natural product for improving adipose mobilization in obesity.

## Introduction

Adipose tissue is a critical organ involved in regulating systemic energy balance and glucose homeostasis [1]. The over-accumulation of lipids within adipose tissue is a major risk factor for chronic diseases including diabetes, cardiovascular diseases, hypertension, and cancer [2–4]. Over the past two decades, extensive research has been conducted on natural anti-obesity substances that may prevent fat storage and accelerate fat disintegration, which may be beneficial in weight control and tackling obesity.

The increase in adipose tissue mass under obese condition is due, at least in part, to an elevation in the number of adipocytes, which is mainly determined by the adipocyte differentiation process, termed adipogenesis [5]. Adipocyte differentiation is controlled by a complex network of transcriptional factors, including members of the peroxisome proliferator-activated receptor-γ (PPARγ) family and CCAAT/enhancer binding protein (C/EBP) [5]. Evidence suggests that extracellular signal-regulated kinase-1/2 (ERK1/2) [6,7] and transforming growth factor-β (TGF-β)/mothers against decapentaplegic homolog 3 (smad3) [8,9] signaling pathways may also be involved in regulating adipogenesis. Strategies that could induce the adipogenesis process could be promising for treating obesity-related disorders.

Lipolysis in white adipose tissue is one of the key mechanisms for maintaining fuel partitioning [10]. Under obese conditions, basal fat cell lipolysis is elevated [11] and is closely associated with insulin resistance and lipotoxicity [12]. Adipocyte lipolysis is also stimulated by catecholamine hormones via elevations in cellular cyclic adenosine monophosphate (cAMP) content and subsequent activation of cAMP-dependent protein kinase A (PKA) [13]. Subsequently, PKA phosphorylates two proteins, hormone-sensitive lipase (HSL) and perilipin, in adipocytes and leads to translocation of HSL from the cytosol to the lipid droplet surface, which is crucial for the lipase to access its triacylglycerol substrates [14,15]. Therefore, inhibition of adipocyte lipolysis may be another promising therapeutic strategy for reducing the level of circulating free fatty acids (FFAs) and improving obesity-associated metabolic dysfunction.

Adipose tissue also functions as an endocrine organ, secreting a wide range of adipokines that regulate lipid and glucose metabolism [16]. For example, leptin has multiple effects such as suppressing food intake, promoting energy expenditure [17], and improving peripheral insulin sensitivity [18]. Adiponectin has been reported to increase glucose uptake in muscles and suppress gluconeogenesis in liver [19]. Resistin was first discovered as an inducer of insulin resistance [20].

Adipose tissue is also a key organ where adipocytes interact with macrophages, T cells, and dendritic cells [21]. During the development of obesity, adipose tissue is infiltrated by an increasing number of macrophages, which are considered to be a major source of inflammatory mediators, such as tumor necrosis factor-α (TNF-α) and nitric oxide, that negatively affect adipocyte function [22].

Rosmarinic acid (RA) is a natural phenol carboxylic acid, which is a secondary metabolite found in plants of the family Lamiaceae that are commonly consumed as culinary herbs, such as lemon balm, rosemary, sage, thyme, and peppermint [23]. RA possesses multiple promising biological effects including anti-cancer [24], anti-microbial, anti-allergic [25], anti-inflammatory, and anti-oxidant properties [26]. In particular, RA may be beneficial in experimental diabetes and hyperlipidemia [27]. Its ability to suppress inflammatory processes and to scavenge oxygen free radicals makes RA a suitable candidate for improving adipose dysfunction in obesity.

With the above points in mind, the aims of the present study are: (i) to explore the effects of RA on two critical processes related to the key functions of adipose tissue, i.e. adipogenesis and lipolysis, and on the messenger RNA (mRNA) expression of major adipokines, i.e. leptin, adiponectin, resistin, and visfatin, using a 3T3-L1 adipocytes model; and (ii) to explore how RA affects lipopolysaccharide (LPS)-induced TNF-α secretion in macrophages, as well as mRNA expression of inflammatory cytokines and chemokines in 3T3-L1 adipocytes incubated with macrophage-conditioned medium (MCM). Findings from this study will help us to explore whether intervention with RA could be an effective strategy for restoring adipose tissue dysfunction under obese conditions.

## Materials and methods

### Materials

The following reagents were obtained from Thermo Fisher Scientific (Waltham, MA, USA): bovine serum albumin (BSA), Dulbecco’s modified Eagle’s medium (DMEM), and trypsin/ethylenediaminetetraacetic acid (EDTA) solution. RA (cat. no. 70900), PD98059 (cat. no. 10006726), forskolin (cat. no. 11018), wortmannin (cat. no. 10010591), rolipram (cat. no. 10011132), cilostamide (cat. no. 14455), and SB431542 (cat. no. 13031) were obtained from Cayman Chemicals (Ann Arbor, MI, USA). Antibodies against phosphorylated (p-) HSLser660 (cat. no. 4126), p-ERK1/2 (cat. no. 4370), p-SMAD3 (9520), SMAD3 (cat. no. 9523), PPARγ (cat. no. 2435), C/EBPα (cat. no. 2843), and β-actin (cat. no. 4970) were from Cell Signaling Technology (Danvers, MA, USA). Anti-perilipin A (cat. no. AB3526) was from abcam (Shanghai, China). Anti-phospho-perilipin S522 was purchased from Vala Sciences (San Diego, CA, USA). Horseradish peroxidase (HRP)-conjugated donkey anti-rabbit and goat anti-mouse immunoglobulin G secondary antibodies were purchased from Jackson ImmunoResearch Laboratories (West Grove, PA, USA). Universal RNA Extraction Kit, PrimeScript™ RT Master Mix kit and Premix Ex Taq™ (Probe qPCR) were from Takara Bio (Shiga, Japan). Taqman Gene Expression Assays for mouse leptin (Mm00440181_m1), adiponectin (Mm00473047_m1), resistin (Mm00445641_m1), visfatin (Mm00451938_m1), TNF-α (Mm00443258_m1), interleukin-6 (IL-6) (Mm01210732_g1), IL-1β (Mm00434228_m1), monocyte chemoattractant protein-1 (MCP-1; Mm00656886_g1), regulated on activation, normal T cell expressed and secreted (RANTES; Mm01302427_m1), and eukaryotic 18S ribosomal RNA (4352930E) were from Applied Biosystems (Foster City, CA, USA). Fatty acid-free BSA (cat. no. BSAS100) was from Bovogen (Melbourne, Australia). Glycerol assay kit (cat. no. E1002) was from Applygen Technologies (Beijing, China). Labassay non-esterified fatty acid (NEFA) kit (cat. no. 294–63601) was purchased from Wako (Osaka, Japan). Mouse TNF-α Duoset enzyme-linked immunosorbent assay (ELISA) kit was from R&D Systems (Minneapolis, MN, USA). Reagents, molecular weight markers, and nitrocellulose membranes for sodium dodecyl sulfate–polyacrylamide gel electrophoresis (SDS-PAGE) were obtained from Bio-Rad (Hercules, CA, USA). All other chemicals were from Sigma (St Louis, MO, USA).

### Cell culture and differentiation of 3T3-L1 adipocytes

Murine 3T3-L1 cells and RAW 264.7 macrophages (ATCC) were cultured in DMEM supplemented with 10% fetal bovine serum (FBS) and 100 units/mL of penicillin/streptomycin in a humidified incubator in 95% air and 5% carbon dioxide. At 2 days post-confluence (designated as day 0), differentiation was induced using basic media supplemented with 1 μmol/L dexamethasone, 0.5 mmol/L isobutylmethylxanthine, and 5 μg/mL insulin for 2 days (day 2). Media were then replaced with basic media supplemented with 5 μg/mL insulin on days 4, 6, and 8 post-differentiation.

### Treatments

#### Adipogenesis

For the detection of adipogenic-related markers during differentiation, cells were collected on days 3, 6, and 9 during differentiation for the measurement of PPARγ and C/EBPα protein expression by Western blotting. For the Oil Red O staining, the 3T3-L1 adipocytes (on day 6 post-differentiation) were cultured in six-well plates and treated with RA in the absence or presence of PD98059 (25 μM) or SB431542 (2 μM) for 24 h. Cells were then washed with phosphate-buffered saline (PBS) and fixed with 10% formalin for 15 min at room temperature. The cells were then washed twice with PBS and stained for 15 min with a filtered Oil Red O staining solution, which consists of Oil Red O (1.8 mg/mL) and isopropanol (60% v/v) in distilled water. This was followed by washing with distilled water. The staining of lipid droplets in adipocytes was observed under an optical microscope. For the measurement of p-ERK1/2 and Smad3, cells were given the same treatment as in the Oil Red O staining experiment. At the end of the treatment, cells were washed twice with cold PBS and lysed on ice with cell lysis buffer containing protease and phosphatase inhibitors for further Western blotting analysis. All of the above experiments were repeated at least three times.

#### Lipolysis

The 3T3-L1 adipocytes (on day 9 post-differentiation) were starved in serum-free DMEM at 37°C for 2 h. For the basal lipolysis experiment, cells were incubated with a wide range of concentrations of RA (50, 100, and 200 μM) or co-incubated with RA (50 μM) in the presence or absence of wortmannin (100 nM), rolipram (5 μM), and cilostamide (5 μM) in DMEM supplemented with 2% fatty acid-free BSA. For stimulated lipolysis experiments, one set of the cells was preincubated with RA (50 μM) for 1 h and lipolysis was stimulated with isoproterenol (ISO) (100 nM) or forskolin (25 mM). Media were collected at indicated time-points (i.e. 2 h and 24 h) for the measurement of glycerol and FFA release. The other set of cells was administered with the same treatment as above, except that cells were collected at 24 h. Thereafter, cells were washed twice with cold PBS and lysed on ice with cell lysis buffer containing protease and phosphatase inhibitors for further Western blotting analysis. All of the experiments were repeated at least three times.

#### Adipokine mRNA expression

The 3T3-L1 adipocytes (on day 9 post-differentiation) were starved in serum-free DMEM at 37°C for 2 h. Cells were incubated with RA (50 μM) for 48 h. Thereafter, cells were washed twice with cold PBS and then RNA was extracted using RNA extraction buffer for further real-time reverse transcription–polymerase chain reaction (RT-PCR) experiments. All of these experiments were repeated at least three times.

#### LPS-stimulated inflammation in macrophages and preparation of MCM

RAW 264.7 macrophages were seeded in 12-well plates at a density of 5 × 10^4^ cells/well and stimulated with LPS (1 ng/mL) in the presence or absence of RA (50 μM) for 24 h for the measurement of TNF-α mRNA expression and secretion. To prepare the MCM, the cells were preincubated with RA (50 μM) for 24 h, then stimulated with LPS (1 ng/mL) for 3 h. The supernatants were then collected and stored at −80°C for further experiments.

### 3T3-L1 adipocytes incubated with MCM

The differentiated 3T3-L1 adipocytes were incubated with MCM for 24 h and cells were collected for further mRNA expression analysis.

### Glycerol and FFA measurement

Culture media were analyzed for FFA and glycerol concentrations using colorimetric assays according to the manufacturer’s instructions. The coefficient of variation for these assays in our laboratory is <10%.

### Western blot analysis

3T3-L1 total cell lysates were prepared in cell lysis buffer supplemented with Protease/Phosphatase Inhibitor Cocktail and phenylmethylsulfonyl fluoride (PMSF; final concentration 100 mM/L). Protein concentrations were determined using a bicinchoninic acid (BCA) assay reagent kit. Changes in the protein expression of PPARγ, C/EBPα, p-ERK1/2, p-Smad3/Smad3, p-HSLser660/HSL, and p-perilipin A/perilipin A were determined by Western blotting as described previously [28]. In brief, proteins were wet transferred to nitrocellulose membranes at 360 mA/tank for 3 h and subsequently blocked in Tris-buffered saline/0.1% Tween 20 (TBST) supplemented with 5% non-fat dry milk for 1 h at room temperature with gentle agitation. Membranes were incubated in TBST/5% non-fat dry milk supplemented with appropriate primary antibodies (dilution 1:1000) overnight at 4°C with gentle agitation. The following morning, membranes were briefly washed in TBST and then incubated in TBST/1% non-fat dry milk supplemented with HRP-conjugated secondary antibodies for 1 h at room temperature. Bands were visualized using Immobilon Western chemiluminescent HRP substrate and captured using a Syngene chemi-imaging system (Frederick, MD, USA). Subsequently, bands were quantified by densitometry using Gene Tools according to the manufacturer’s instructions (Syngene ChemiGenius2, PerkinElmer). Protein contents of phosphorylated protein (i.e. p-ERK1/2, p-Smad3, p-HSLser660, and p-perilipin A) were quantified and normalized to the total levels of these proteins. βActin was used as an internal loading control for quantification of all other proteins.

### Real-time RT-PCR

RNA was isolated from differentiated 3T3-L1 adipocytes or RAW 264.7 macrophages using an RNeasy kit (CW Biotechnology, Beijing, China) according to the manufacturer’s instructions. One microgram of RNA was used for the synthesis of complementary DNA (cDNA) using PrimeScript RT master mix containing Primerscript RTase, oligo(dT), and dNTP. Real-time PCR was performed using a QuantStudio6 Standard96 Real-Time PCR system (Applied Biosystems, Foster City, CA, USA). Results were normalized to the mRNA expression of 18S. Relative differences in gene expression between groups were determined using the 2^−∆∆CT^ method [29]. The amplification efficiencies of the gene of interest and the housekeeping gene were equivalent.

### Statistical analysis

All data are presented as means ± SEM. Comparisons between groups were analyzed using one-way analysis of variance (ANOVA) followed by a Tukey’s *post hoc* test for multiple comparisons. The level of statistical significance was set at *p* < 0.05.

## Results

### RA suppressed adipogenesis

**Figure 1.**
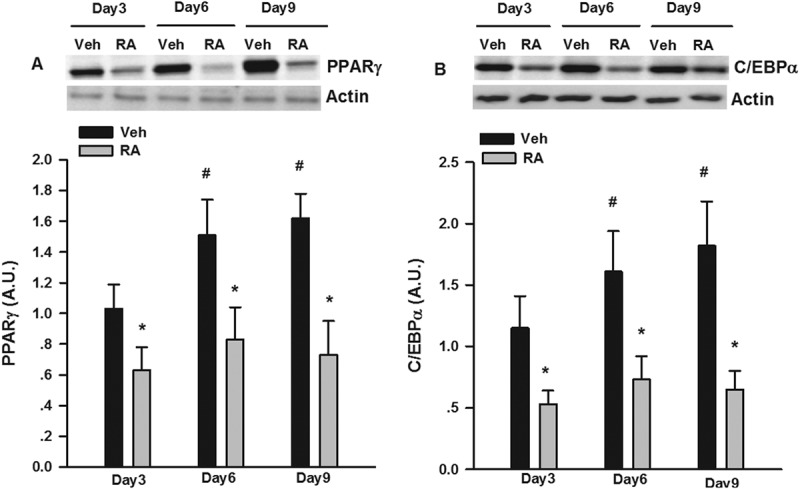
Suppression by rosmarinic acid (RA) of peroxisome proliferator-activated receptor-γ (PPARγ) and CCAAT/enhancer binding protein-α (C/EBPα) protein expression during the differentiation of 3T3-L1 adipocytes. Cells were collected on days 3, 6, and 9 during differentiation for the measurement of PPARγ and C/EBPα protein expression by Western blotting. Representative blots are shown above the graphs. Data are presented as means ± SEM. All cell culture experiments were repeated at least three times on two separate passages of cells (*n* = 6). *p < 0.05 vs vehicle (Veh) group at the same time-point; #*p* < 0.05 vs RA group on day 3 of differentiation.

**Figure 2.**
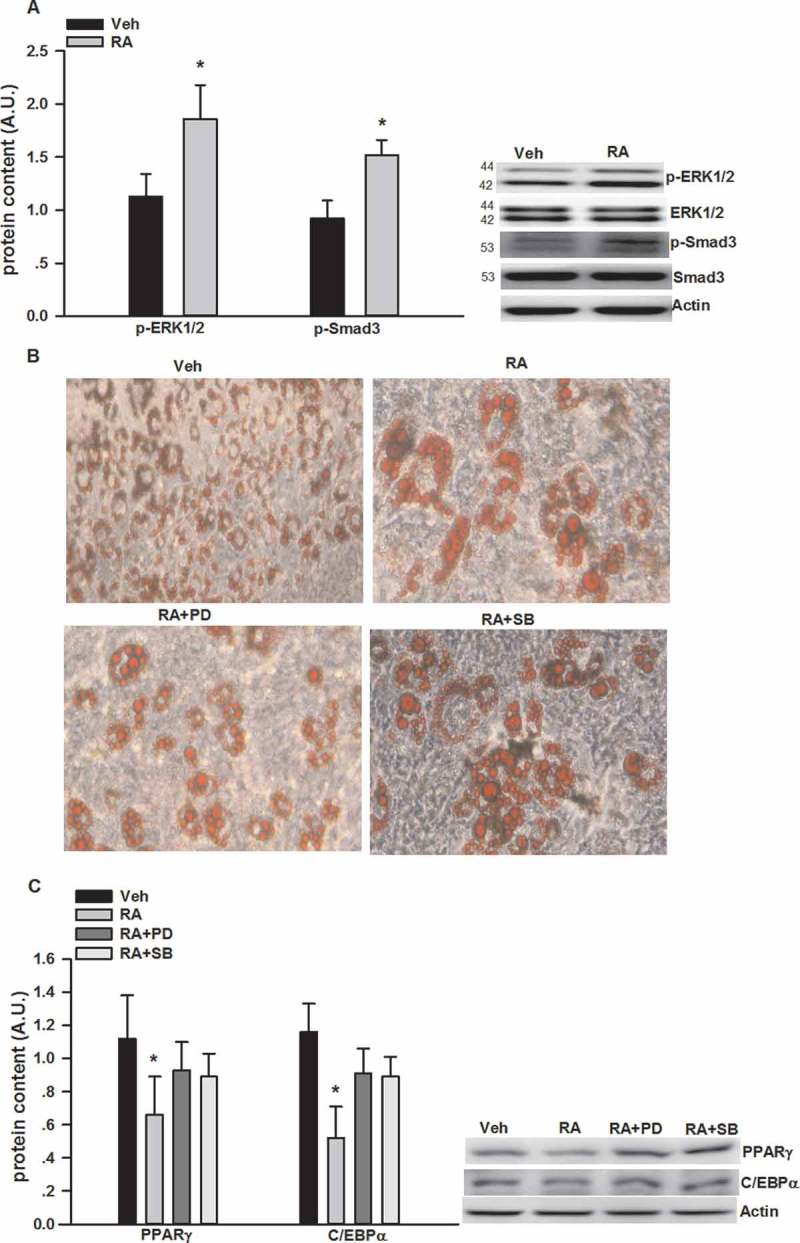
Inhibition of adipogenesis by rosmarinic acid (RA) via the activation of extracellular signal-regulated kinase-1/2 (ERK1/2) and mothers against decapentaplegic homolog 3 (Smad3). (A) Protein expression of phosphorylated (p-)ERK1/2 and Smad3 after RA (50 μM, 12 h) incubation on day 6 of differentiation. (B) Oil Red O staining of the 3T3-L1 adipocytes (on day 6 post-differentiation) treated with RA in the absence or presence of PD98059 (PD, 25 μM) and SB431542 (SB, 2 μM) for 24 h. (C) Protein expression of p-ERK1/2 and Smad3 after RA (50 μM, 12 h) incubation in the presence or absence of PD98059 (25 μM) and SB431542 (2 μM) for 24 h. Representative blots are shown to the right of the graphs. Data are presented as means ± SEM in (A) and (C). All cell culture experiments were repeated at least three times on two separate passages of cells (*n* = 6). *p < 0.05 vs vehicle (Veh) group.

PPARγ and C/EBPα are two critical factors involved in adipogenesis [30]; thus, we measured PPARγ and C/EBPα protein expression at different stages of 3T3-L1 differentiation. As shown in Figure 1, the expression of PPARγ and C/EBPα significantly increased as the 3T3-L1 cells differentiated into adipocytes. RA significantly suppressed the progressive expression of PPARγ and C/EBPα protein expression. As shown in Figure 2(A), RA led to about two-fold induction in p-ERK1/2 and p-Smad3 protein expression at day 6 of differentiation. We also used PD98059 and SB431542, well-established inhibitors of p-ERK1/2 [31] and p-Smad3 [32] signaling pathways, respectively, to explore whether they could reverse RA inhibition of adipogenesis. Both PD98059 and SB431542 partially rectified the inhibitory effects of RA on the number of lipid-droplet containing adipocytes via Oil Red O staining (Figure 2(B)). Both PD98059 and SB431542 also partially reversed the expression of adipogenic transcriptional factors, i.e. PPARγ and C/EBPα, respectively, measured by Western blotting (Figure 2(C)).

### RA inhibited basal lipolysis

As shown in Table 1, RA (50, 100, and 200 μM, 24 h) inhibited glycerol and FFA release, while no dose–response effects were observed.

**Table 1.** Effects of rosmarinic acid (RA) on basal glycerol and free fatty acid (FFA) release (μm/L).

	Vehicle	RA50	RA100	RA200
Glycerol	84.43 ± 7.08	63.15 ± 10.8*	57.64 ± 13.2*	50.6 ± 9.4*
FFAs	187.2 ± 8.51	93.7 ± 4.13*	92.2 ± 2.41*	116.8 ± 3.03*

Data are presented as mean ± SEM.

Glycerol and FFA release was measured in cultured 3T3-L1 adipocytes incubated with several concentrations of RA (50, 100, and 200 μM) in Dulbecco’s modified Eagle’s medium supplemented with 2% fatty acid-free bovine serum albumin for 24 h.

*p < 0.05 vs vehicle group.

### RA inhibited basal lipolysis via phosphodiesterase-3 (PDE3)

**Figure 3.**
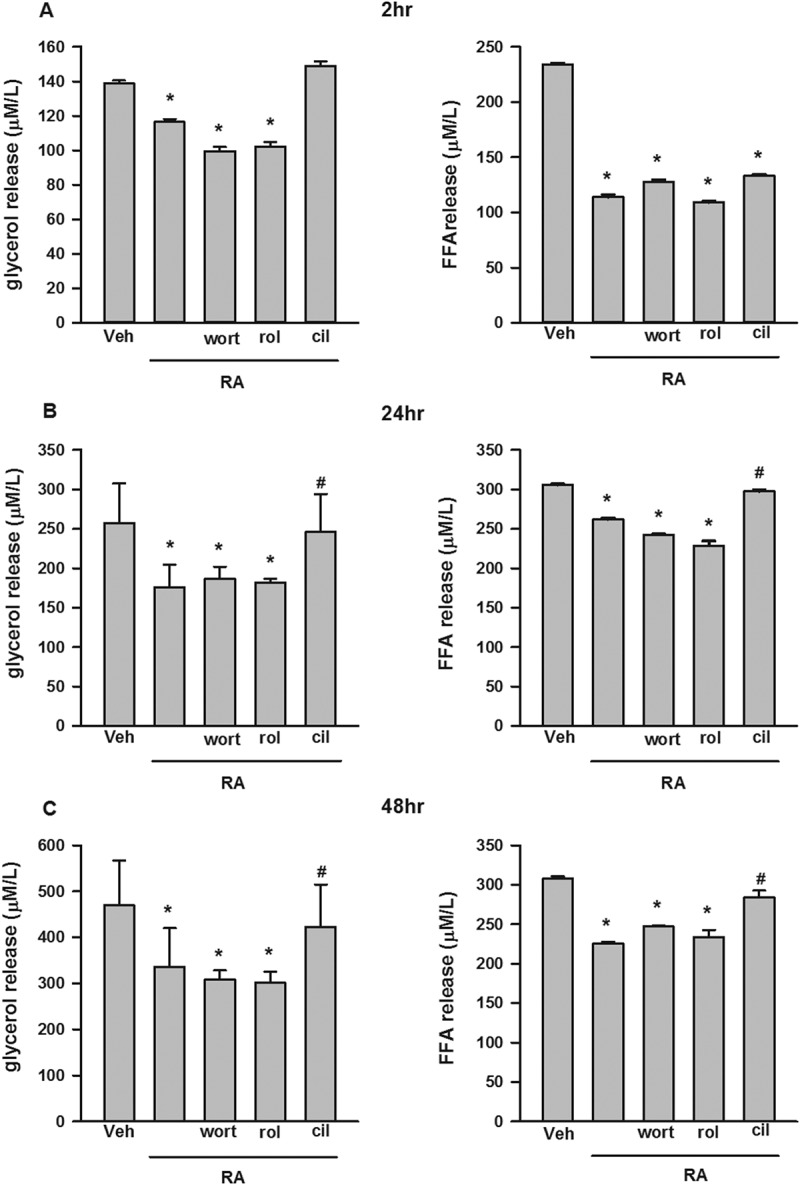
Glycerol and free fatty acid (FFA) release in cultured 3T3-L1 adipocytes incubated with rosmarinic acid (RA) in the presence or absence of wortmannin (wort, 100 nM), rolipram (rol, 5 μM), and cilostamide (cil, 5 μM) in Dulbecco’s modified Eagle’s medium supplemented with 2% fatty acid-free bovine serum albumin at 2 h (A), 24 h (B), and 48 h (C). Data are presented as means ± SEM. All cell culture experiments were repeated at least three times on two separate passages of cells (*n* = 6). *p < 0.05 vs vehicle (Veh) group at the same time-point; #*p* < 0.05 vs RA group at the same time-point.

Wortmannin, cilostamide, and rolipram, which are phosphatidylinositol 3-kinase (PI3K), PDE3, and specific PDE4 inhibitors [33–35], respectively, were used to test whether RA-mediated anti-lipolytic effects took place via a similar pathway to insulin [36]. As shown in Figure 3(A), 2 h post-treatment, compared to the vehicle group, the glycerol release from the RA, RA/wortannin, and RA/rolipram groups, and the FFA release from the RA, RA/wortannin, RA/rolipram, and RA/cilostamide groups were significantly reduced. At 24 h and 48 h, compared to the vehicle group, the glycerol and FFA release from the RA, RA/wortannin, and RA/rolipram groups were still significantly reduced; meanwhile, compared to the RA group, the glycerol and FFA release in the RA/cilostamide group were significantly elevated (Figure 3(B,C)).

### p-HSLser660 and p-perilipin may be involved in RA inhibition of stimulated lipolysis

**Figure 4.**
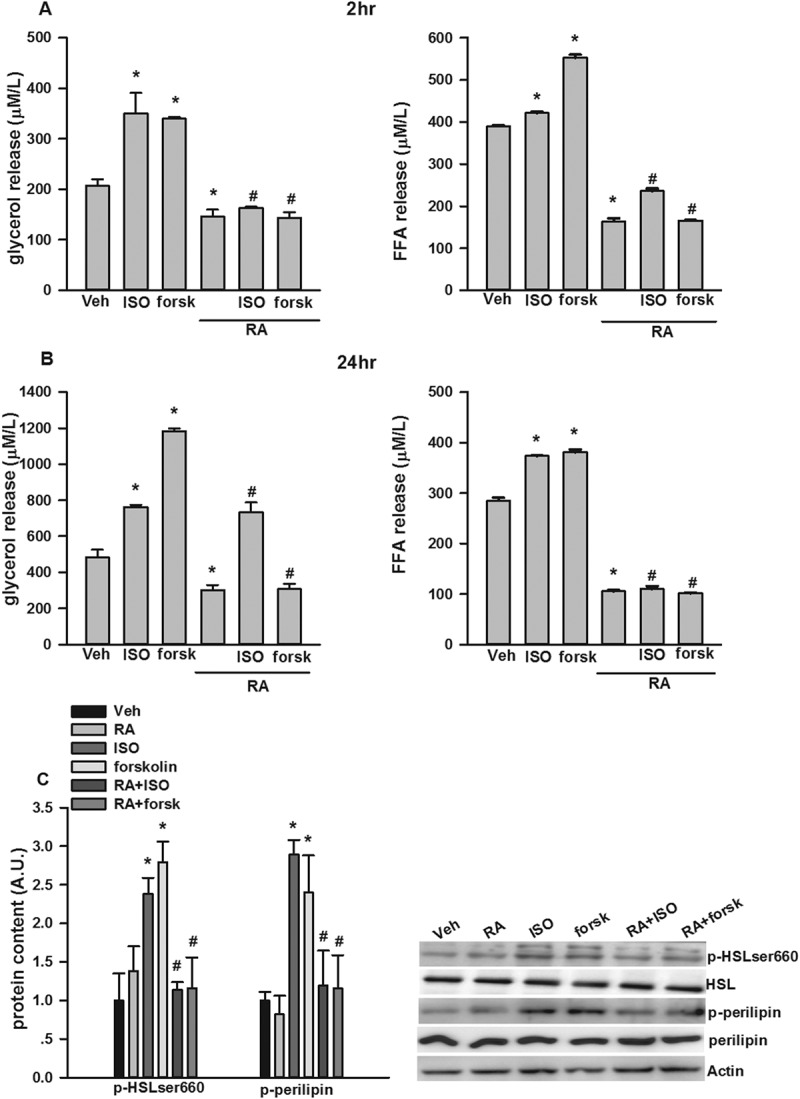
Inhibition by rosmarinic acid (RA) of isoproterenol (ISO)- and forskolin (forsk)- stimulated glycerol and free fatty acid (FFA) release in cultured 3T3-L1 adipocytes. RA suppressed ISO- and forsk-stimulated glycerol and FFA release at both 2 h (A) and 24 h (B). RA inhibited ISO- and forsk-induced phosphorylation of hormone-sensitive lipase (HSL)ser660 and perilipin A (C). Representative blots are shown to the right of the graphs in (C). Data are presented as means ± SEM. All cell culture experiments were repeated at least three times on two separate passages of cells (*n* = 6). *p < 0.05 vs vehicle (Veh) group at the same time-point; #*p* < 0.05 vs RA group at the same time-point.

ISO and forskolin were used to increase cAMP concentrations by different mechanisms, leading to activation of PKA and HSL, together with other proteins stimulating lipolysis [37]. As shown in Figure 4(A,B), ISO and forskolin significantly stimulated glycerol and FFA release at both 2 h and 24 h. The addition of RA significantly inhibited ISO- and forskolin-stimulated glycerol and FFA release at both 2 h and 24 h. We confirmed the results by measuring key proteins involved in lipolysis. As shown in Figure 4(C), ISO and forskolin significantly stimulated the phosphorylation of HSL at ser660 and perilipin A. RA reversed elevated p-HSLser660 and p-perilipin A stimulated by ISO and forskolin.

### RA had no effect on leptin, adiponectin, resistin, or visfatin mRNA expression

There was no significant difference in leptin, adiponectin, resistin, and visfatin mRNA expression between vehicle and RA-treated wells (fold change: 1.3 ± 0.13, 0.89 ± 0.17, 1.09 ± 0.07, and 1.02 ± 0.07, respectively).

### RA suppressed TNF-α mRNA expression and secretion in LPS-stimulated RAW 264.7 macrophages

**Figure 5.**
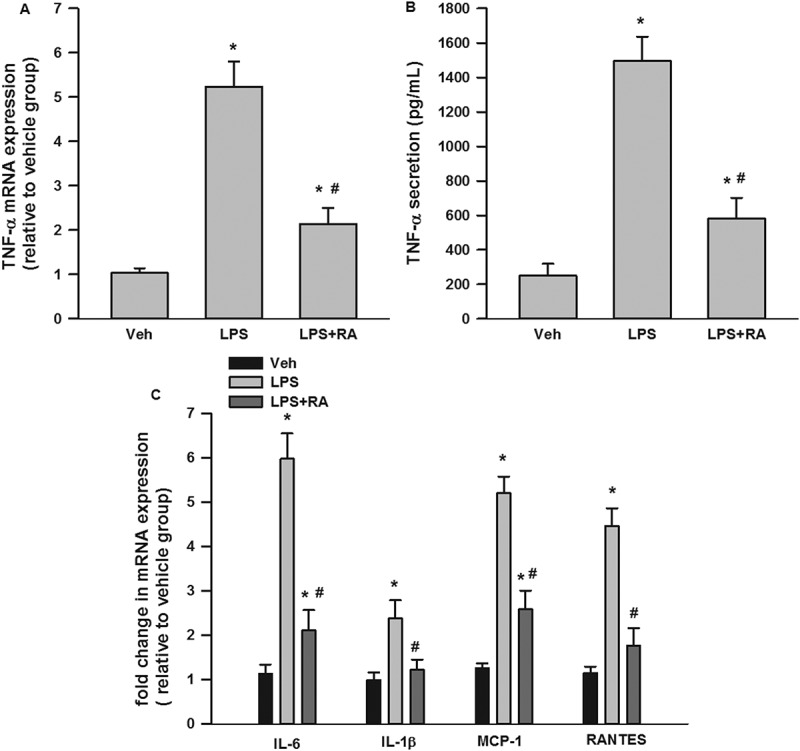
Suppression by rosmarinic acid (RA) of lipopolysaccharide (LPS)-stimulated tumor necrosis factor-α (TNF-α) messenger RNA (mRNA) expression and secretion in RAW 264.7 macrophages, and inflammatory cytokine mRNA expression in 3T3-L1 adipocytes stimulated with LPS-macrophage-conditioned medium (MCM). RA inhibited LPS-stimulated TNF-α mRNA expression (A) and secretion (B) in RAW 264.7 macrophages. RA inhibited interleukin-6 (IL-6), IL-1β, monocyte chemoattractant protein-1 (MCP-1), and regulated on activation, normal T cell expressed and secreted (RANTES) mRNA expression in 3T3-L1 adipocytes incubated with LPS-MCM (C). *p < 0.05 vs vehicle (Veh) group; #*p* < 0.05 vs LPS group (in A and B); #*p* < 0.05 vs LPS-MCM group (in C).

As shown in Figure 5(A), LPS significantly induced TNF-α mRNA expression (about five- fold) and RA significantly inhibited LPS-stimulated TNF-α mRNA expression. Similarly, LPS also significantly stimulated TNF-α secretion (approximately seven-fold) and RA suppressed LPS-induced TNF-α secretion (Figure 5(B)).

### RA reduced MCM-induced proinflammatory cytokine and chemokine mRNA expression in 3T3-L1 adipocytes

To explore how RA-treated macrophages affect adipocytes, 3T3-L1 adipocytes were incubated with MCM for 24 h. We evaluated mRNA expression of major inflammatory genes including IL-6, IL-1β, MCP-1, and RANTES. As shown in Figure 5(C), the mRNA expression of IL-6, IL-1β, MCP-1, and RANTES in 3T3-L1 adipocytes following LPS-MCM incubation was significantly increased compared to vehicle-MCM incubation; as expected, RA preincubation partly reduced the elevated IL-6 and MCP-1, and completely suppressed IL-1β and RANTES mRNA expression in 3T3-L1 adipocytes.

## Discussion

**Figure 6.**
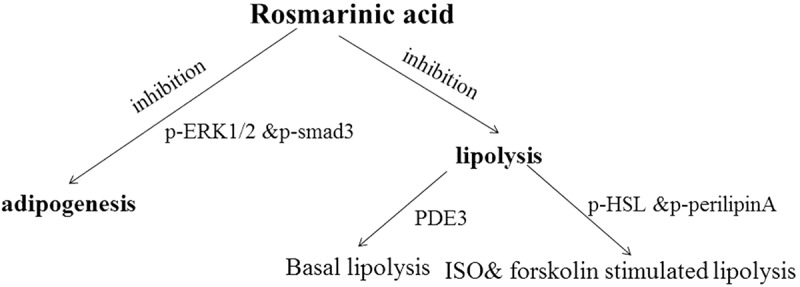
Brief summary of the metabolic networks involved in the effects of rosmarinic acid (RA) on adipogenesis and lipolysis. RA inhibits adipogenesis via phosphorylated extracellular signal-regulated kinase-1/2 (p-ERK1/2) and mothers against decapentaplegic homolog 3 (p-Smad3), basal lipolysis via phosphodiesterase-3 (PDE3), and isoproterenol (ISO)- and forskolin-stimulated lipolysis via hormone-sensitive lipase (p-HSL) and p-perilipin A.

The primary findings of the current study are that (i) ERK1/2 and Smad3-mediated signaling pathways may be involved in the inhibition of adipogenesis by RA; (ii) RA inhibited basal lipolysis via activation of PDE3, and RA also suppressed ISO- and forskolin-stimulated lipolysis, accompanied by RA inhibition of the phosphorylation of HSL and perilipin A; and (iii) RA suppressed LPS-stimulated TNF-α secretion in macrophages, as well as mRNA expression of inflammatory mediators including IL-6, IL-1β, MCP-1, and RANTES in 3T3-L1 adipocytes exposed to MCM. A brief summary of the metabolic networks involved in the effects of RA on adipogenesis and lipolysis is shown in Figure 6.

It has been well documented that PPARγ and C/EBPα function sequentially and cooperatively in stimulating fat cell differentiation [30]. PPARγ is essential for adipogenesis and C/EBPα is critical for maintaining the expression of PPARγ [38]. The present study demonstrated that RA could suppress adipogenesis by the down-regulation of PPARγ and C/EBPα. TGF-β/Smad3 [8,9] and ERK1/2 [6,7] may also be putative mediators of adipogenesis. For example, activation of ERK1/2 signaling is required for preadipocyte factor 1 inhibition of adipogenesis [6]. Sustained activation of ERK1/2 during the adipocyte differentiation process suppresses adipogenesis [7]. Similarly, Choy et al. [8] first reported that dominant negative inhibition of Smad3 enhanced adipogenesis in 3T3-F442A cells. Tsurutani et al. [9] also confirmed the critical role of TGF-β/Smad3 signaling in the inhibition of adipogenesis in Smad3 knockout mice. The present study demonstrated for the first time that ERK1/2 and Smad3 signaling are also involved in the inhibition of adipogenesis by RA. This is evidenced by (i) the activation of ERK1/2 and Smad3 following RA incubation, and (ii) the restoration of adipogenesis after treatment with ERK1/2 and Smad3 inhibitors when incubated together with RA. Previously, it was reported that RA and *Rosmarinus officinalis* polyphenols increased p-ERK1/2 in hippocampal cells [39] and rat pheochromocytoma PC12 cells [40], which is consistent with the present study. Kim et al. [41] reported that *Elsholtzia ciliata* (Thunb.) Hylander ethanol extract (ECE), which contains high amounts of luteolin and RA, blocked the activation of TGF-β/Smad3 signaling in the kidney, which is in contrast to the present study in regard to Smad3 signaling post-RA treatment. This indicates that RA may affect Smad3 signaling in a tissue-specific manner. Considering the critical potential role of Smad3 signaling in obesity [42], further studies are required to explore whether RA could positively affect adipose tissue function under obese conditions.

Catecholamines stimulate adipocyte lipolysis by binding to β-adrenoceptors, resulting in an increase in intracellular cAMP and activation of PKA. PKA then phosphorylates both perilipin and HSL [13]. The phosphorylation of HSL leads to an elevation in hydrolytic activity of the enzyme and the translocation of HSL from the cytosol to the lipid droplet [13–15]. In contrast, insulin is a major anti-lipolytic hormone under basal conditions; this action is mediated mainly through the inhibition of the above cAMP-dependent pathway by phosphorylation of PDE3B, which consequently hydrolyzes cAMP to AMP [36]. Impaired insulin inhibition of basal lipolysis has been observed in enlarged mature adipocytes [43], and elevated levels of circulating FFAs could result in decreased glucose utilization in muscle cells and stimulate hepatic glucose production [44]. The present study suggested that RA could also inhibit basal lipolysis via PDE3, through a signaling pathway that is similar to insulin. We also found that RA could suppress ISO- and forskolin-stimulated lipolysis; this is mediated, at least in part, via its inhibitory effects on the phosphorylation of HSL and perilipin. Collectively, our study provides the first direct evidence that the anti-lipolytic action of RA in adipocytes may allow this phytochemical to limit the concentration of circulating FFA levels, which could be extremely beneficial in pathologies such as obesity and type 2 diabetes. However, further studies are required to elucidate whether RA could suppress lipolysis *in vivo*, as well as whether similar signaling pathways to those observed in the present study are involved.

The significant roles of adipokines in regulating lipid and glucose metabolism [16], as well as in regulating adipose tissue biology *per se* [45], have been greatly appreciated. Previously, phytochemicals such as resveratrol [46] and anthocyanins [47] have been reported to affect the mRNA expression of multiple adipokines. However, we observed no effect of RA on leptin, apelin, resistin, or visfatin mRNA expression in cultured 3T3-L1 adipocytes. Nevertheless, it is likely that (i) RA affects adipokines other than those measured in the present study; and (ii) RA affects leptin, adiponectin, resistin, and visfatin secretion via post-translational mechanisms. Further studies are required to elucidate these points.

TNF-α has been considered to be the key mediator in the deleterious paracrine loop between adipocytes and macrophages [48]. Lin et al. [49] reported that ethanolic extract of *Muntingia calabura* Linn. fruit, which contains RA, suppressed LPS-stimulated proinflammatory cytokines, including TNF-α, IL-6, and IL-1β, in RAW 264.7 macrophages. Our study is the first to report the inhibition by RA of TNF-α mRNA expression and secretion in macrophages in the context of adipose tissue metabolism. We further reported that MCM stimulated with LPS induced inflammatory mediators in 3T3-L1 adipocytes, including IL-6, IL-1β, MCP-1, and RANTES, and RA could partially reverse this situation. Taking these results together, it is suggested that RA may inhibit macrophage inflammation and insulin resistance in adipocytes.

In conclusion, RA demonstrated notable inhibitory effects on adipogenesis, lipolysis, and inflammation, consequently reducing the capacity to produce mature adipocytes from precursor cells and regulating adipose mass, as well as reducing circulating FFAs and inflammatory mediators. Therefore, RA could be a promising natural product for improving adipose mobilization in obesity-related disorders. However, in-depth *in vivo* studies in various animal models, followed by studies in humans, are required to further elucidate the potential applications of RA as a therapeutic agent for obesity.
